# Supplementary material for: Polo-like kinase phosphorylation of the orphan kinesin KIN-G negatively regulates centrin arm biogenesis in Trypanosoma brucei
Source: eLife. 2026 Sep 4;15:RP110793. doi: 10.7554/eLife.110793 (PMC13544905; doi:10.7554/eLife.110793)
Supplement: Supplementary file 1 [file elife-110793-fig1-data1.pdf]

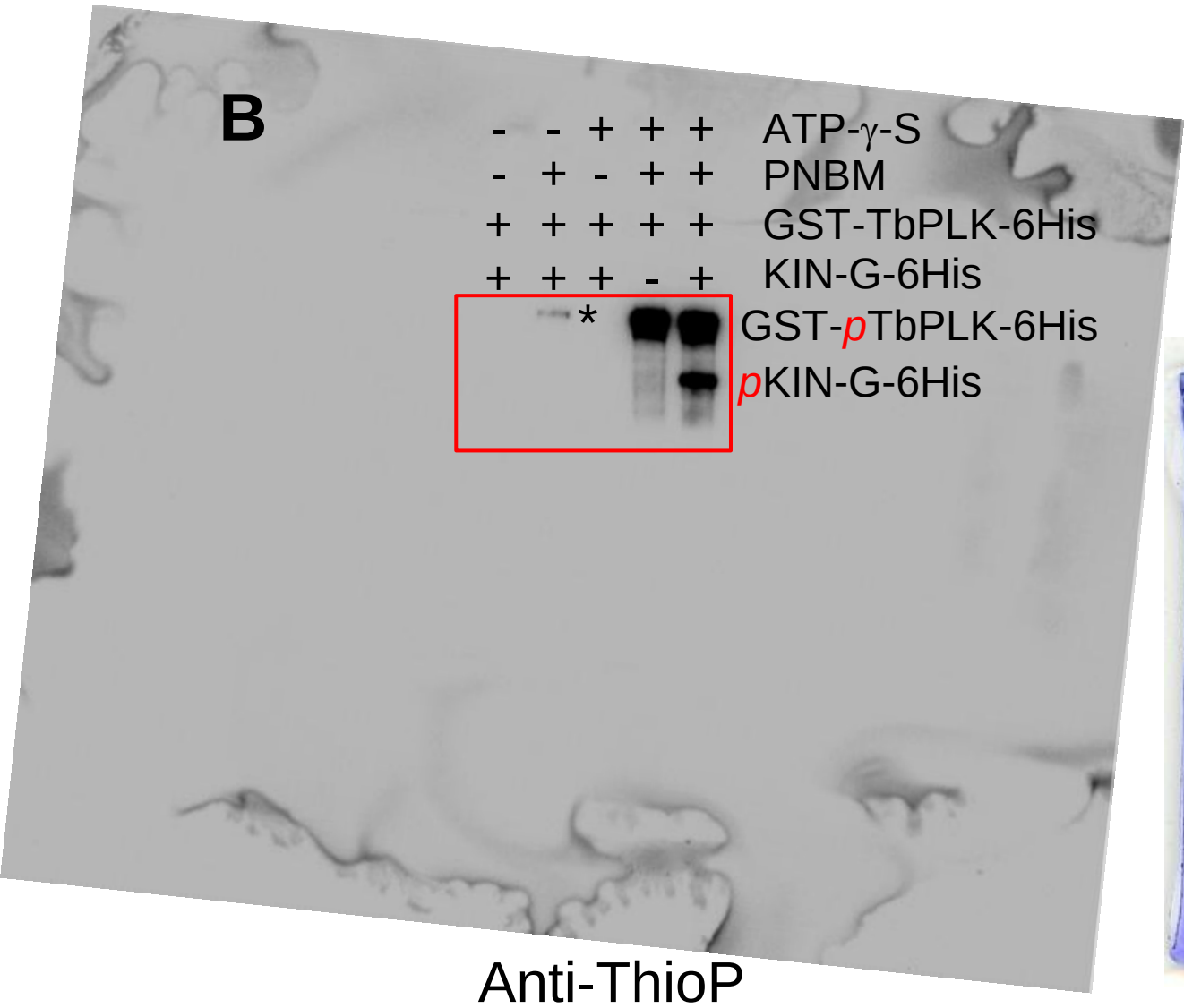

Anti-ThioP

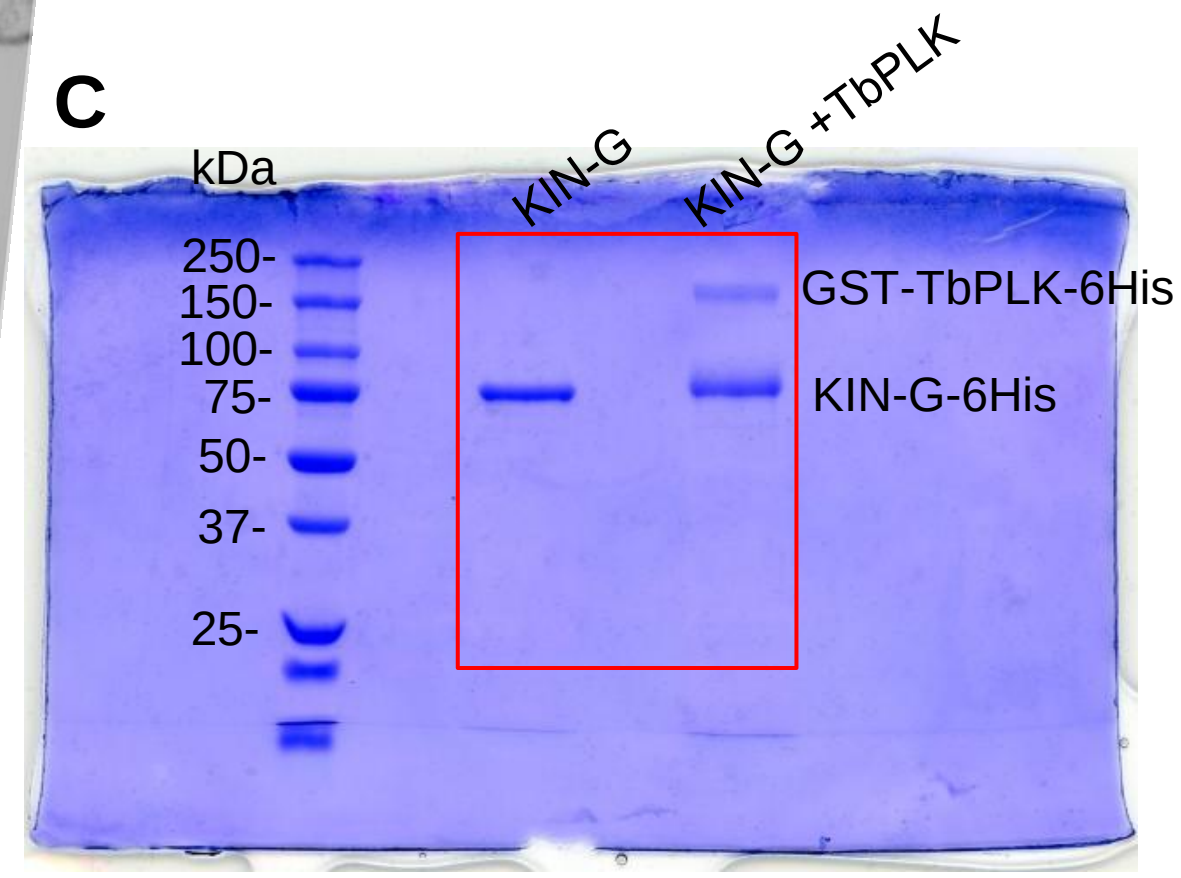

Coomassie blue staining

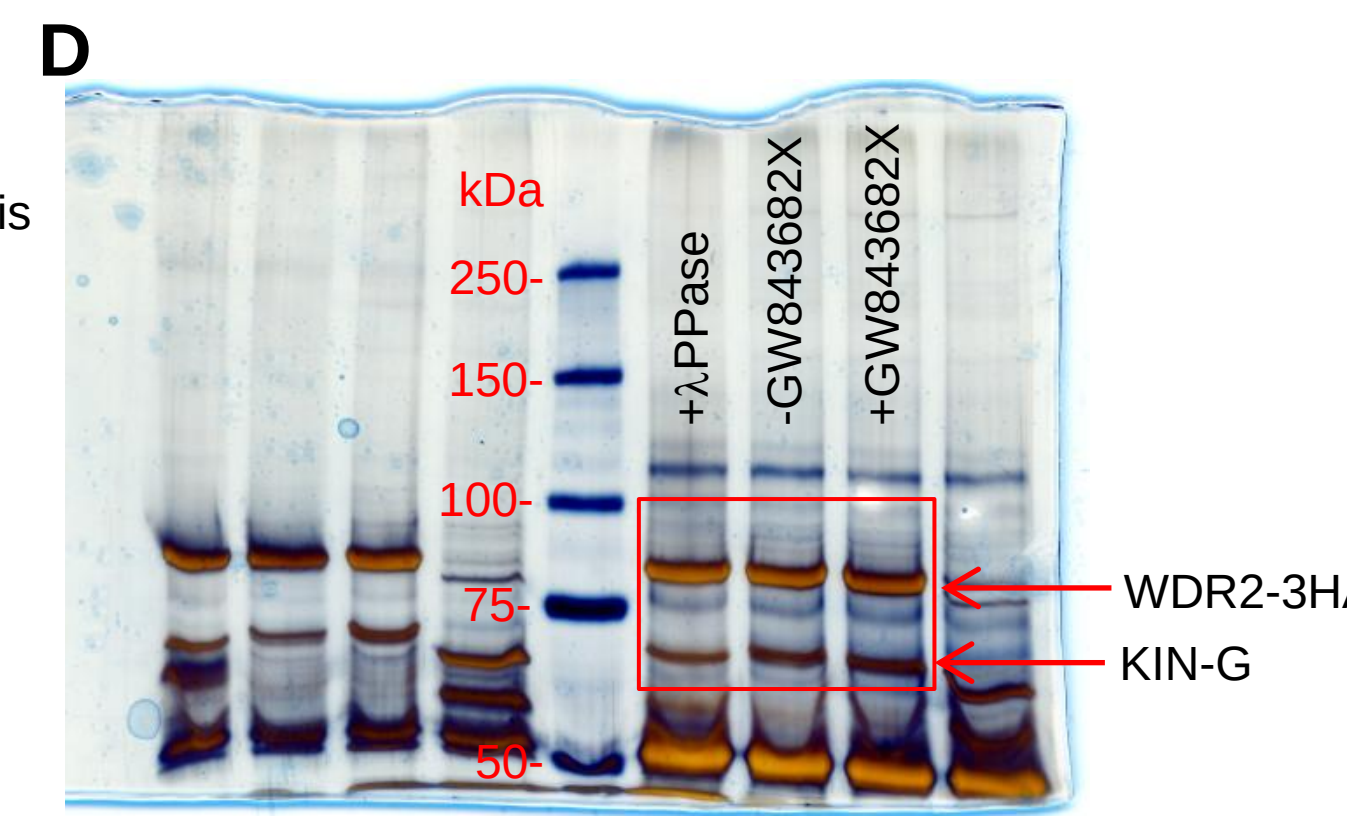

Silver staining

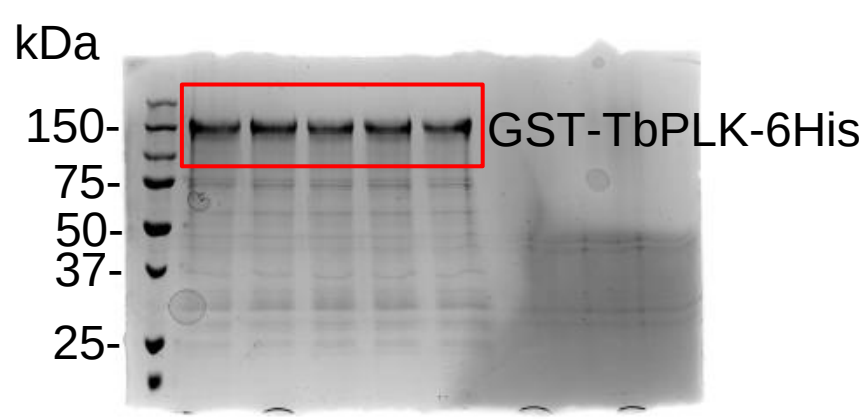

Coomassie blue staining

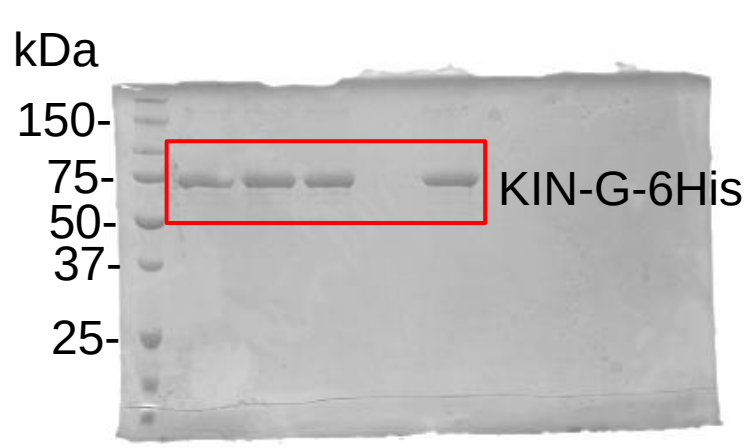

Coomassie blue staining

Figure 1 - source data 1. Original western blots, Coomassie-stained gels and silver-stained gels, corresponding to Figure 1, panel B, panel C, and panel D.
