# Supplementary material for: Polo-like kinase phosphorylation of the orphan kinesin KIN-G negatively regulates centrin arm biogenesis in Trypanosoma brucei
Source: eLife. 2026 Sep 4;15:RP110793. doi: 10.7554/eLife.110793 (PMC13544905; doi:10.7554/eLife.110793)
Supplement: Supplementary file 2 [file elife-110793-fig4-data1.pdf]

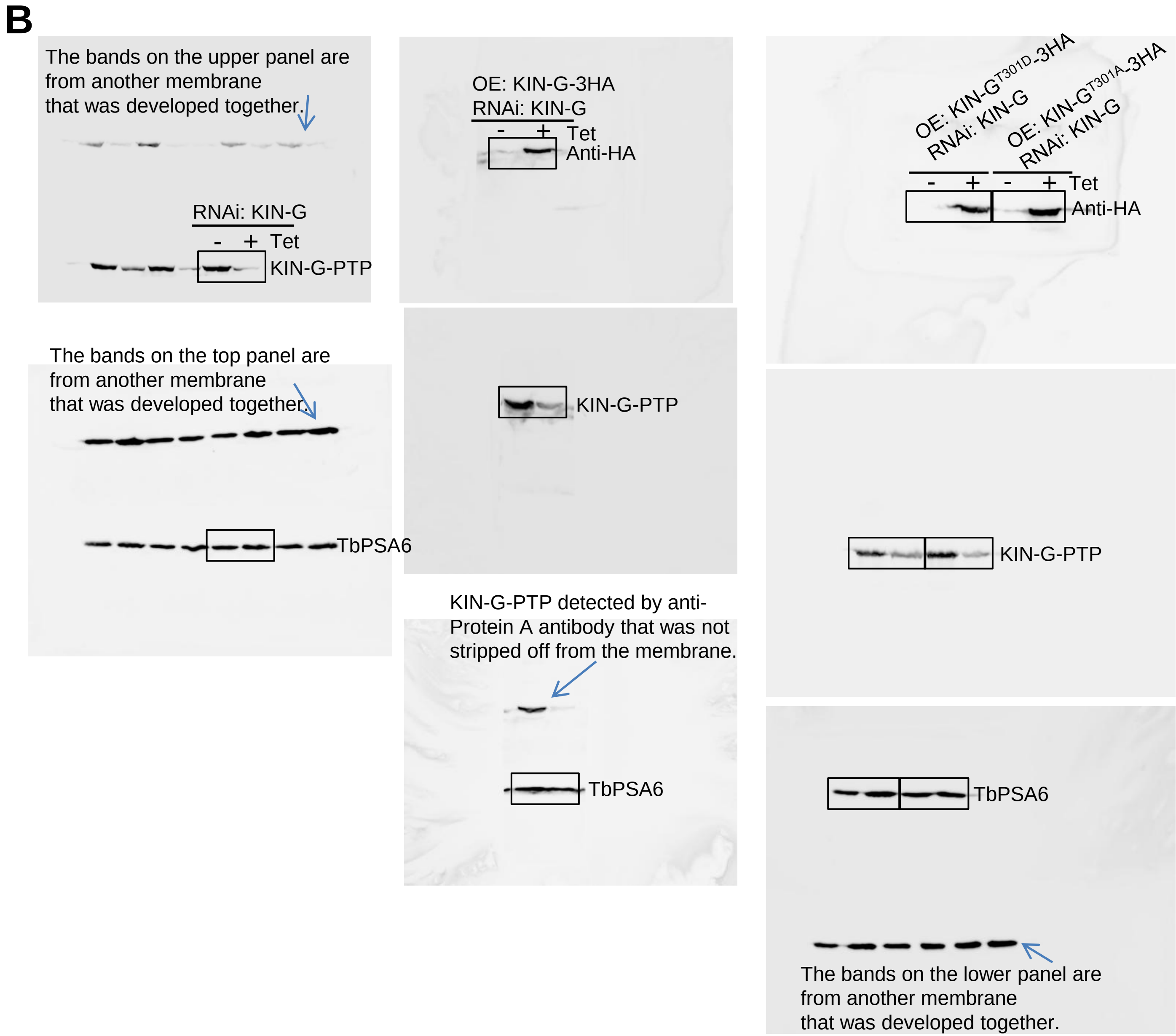

Figure 4 - source data 1. Original western blots corresponding to Figure 4, panel B.
