# Supplementary material for: Polo-like kinase phosphorylation of the orphan kinesin KIN-G negatively regulates centrin arm biogenesis in Trypanosoma brucei
Source: eLife. 2026 Sep 4;15:RP110793. doi: 10.7554/eLife.110793 (PMC13544905; doi:10.7554/eLife.110793)
Supplement: Peer review history

# Editor report: eLife Assessment

**DOI:** 10.7554/eLife.110793.4.sa0

**Authors:** Dominique Soldati-Favre

**Affiliations:** University of Geneva Switzerland

**Keywords:** Convincing, Important

This **important** study provides new insights into the regulation of cell organization and division in *Trypanosoma brucei* through the phosphorylation-dependent control of a kinesin motor protein by a polo-like kinase. The authors present **convincing** evidence, combining rigorous biochemical, cell biological, and imaging analyses, demonstrating that phosphorylation modulates kinesin biochemical activity and molecular function, thereby influencing cellular organization and cytokinesis. The findings advance our understanding of the molecular mechanisms governing trypanosome cell division and will be of broad interest to researchers studying trypanosomes, cytoskeletal regulation, and eukaryotic cell division.

# Referee report: Reviewer #1 (Public review)

**DOI:** 10.7554/eLife.110793.4.sa1

[Editors' note: this version has been assessed by the Reviewing Editor without further input from the original reviewers.]

I think the main points raised in the review have now been addressed. In particular, the new experiment with TbPLK inhibition and mass spectrometry is an important addition, as it provides direct evidence that phosphorylation of KIN-G at Thr301 and Ser569 depends on TbPLK activity in cells.

I also appreciate that the authors have toned down the interpretation of the Golgi phenotype. The revised text now makes clear that the fluorescence data show altered Golgi/ERES organization or duplication, but do not prove a structural defect in Golgi biogenesis.

The added discussion of the T301A result is also helpful. The finding that only a small fraction of KIN-G is phosphorylated at Thr301 in asynchronous cells makes the lack of a strong T301A phenotype more understandable.

Overall, I am happy with the revision of the beautiful manuscript.

# Referee report: Reviewer #2 (Public review)

**DOI:** 10.7554/eLife.110793.4.sa2

Summary:

The authors identify KIN-G as an in vitro substrate for phosphorylation by TbPLK and show that several of the in vitro P-ated sites, including T310, overlap with P-ation sites seen in live cells. The authors further show that PLK-mediated P-ation inhibits KIN-G binding to microtubules in vitro, as does a KIN-G-T301D mutant, and that expression of a KIN-G-T301D Phospho-mimic in *T. brucei* phenocopies KIN-G RNAi knockdowns, producing defects in cell division, morphogenesis of the centrin arm, FAZ and other cellular structures, as well as misplaced cytokinesis furrow.

Understanding cytoskeletal rearrangements that drive cell division in *T. brucei* is an important and unresolved problem, so the work addresses important questions that are of great interest. PLK and KIN-G have previously been shown to be important for cell division and morphogenesis of cytoskeletal structures that drive cell division in *T. brucei*. The current work advances our understanding by suggesting a potential mechanism by which PLK and KIN-G might participate, namely through PLK-dependent P-ation to control KIN-G MT binding activity.

Strengths:

The authors use a rigorous combination of biochemistry, phosphoproteomics, cell biology, and mutant analysis to support their conclusion that PLK-mediated P-ation of KIN-G negatively regulates KIN-G microtubule binding and this may explain the observation that a KIN-G T301 phosphomimic mutant blocks cell division and perturbs biogenesis of cytoskeletal structures that drive cell division and morphogenesis. Combining rigorous and informative in vitro studies with mutant analysis in live cells is a great strength. The work is solid and important, though a few pieces are needed to fully connect the in vitro findings with the in vivo observations.

# Referee report: Reviewer #3 (Public review)

**DOI:** 10.7554/eLife.110793.4.sa3

Summary:

Here the authors investigate the role of the *Trypanosoma brucei* polo-like kinase TbPLK in the function of flagellum-associated cellular structures in trypanosomes. They set out to test the hypothesis that a key substrate of TbPLK is the kinesin protein KIN-G, and that TbPLK phosphorylation of KIN-G regulates its functions in cells.

Strengths:

Using in vitro biochemistry with purified proteins, the authors convincingly demonstrate that TbPLK phosphorylates KIN-G at 29 sites. Moreover, they convincingly show that phosphorylation at one site, T301, impairs the binding of purified KIN-G to purified microtubules. They further confirm that inhibition of TbPLK in cells reduces KIN-G phosphorylation at T301 (and S569). Using immunofluorescence-based imaging approaches, they also show that TbPLK colocalizes with KIN-G at centrin arms during early S-phase of the cell cycle. Centin arms are structures that are located near the basal body and flagellum and are important for new flagellum biogenesis, Golgi positioning, and cell division. To evaluate the function of KIN-G phosphorylation in cells, they depleted KIN-G by RNAi, simultaneously expressed phospho-mimetic (T301D) and phospho-ablative mutant proteins, and used immunofluorescene to examine the impact on flagellum-associated cellular structures. They show that expression of the phospho-mimetic mutant KIN-G-T301D causes the following defects: reduced cell proliferation, disruption of centrin arm and Golgi biogenesis, impairment of FAZ elongation and flagellum positioning, and misplacement of the cell division plane. The data convincingly support the conclusion that KIN-G phosphorylation on T301 plays an important role in regulating the cellular functions of this kinesin motor protein.

# Author comment: Author response

**DOI:** 10.7554/eLife.110793.4.sa4

**Authors:** Yasuhiro Kurasawa, Qing Zhou, Kyu Joon Lee, Huiqing Hu, Ziyin Li

**Affiliations:** The University of Texas Health Science Center at Houston Houston United States; University of Texas Medical School at Houston Houston United States; Soon Chun Hyang University Asan Republic of Korea; University of Texas Medical School at Houston Houston United States; The University of Texas Health Science Center at Houston Houston United States

The following is the authors’ response to the previous reviews

**Public Reviews:**

**Reviewer #1 (Public review):**

I thank the authors for the revised manuscript and for the detailed responses.

I think the main points raised in the review have now been addressed. In particular, the new experiment with TbPLK inhibition and mass spectrometry is an important addition, as it provides direct evidence that phosphorylation of KIN-G at Thr301 and Ser569 depends on TbPLK activity in cells.

I also appreciate that the authors have toned down the interpretation of the Golgi phenotype. The revised text now makes clear that the fluorescence data show altered Golgi/ERES organization or duplication, but do not prove a structural defect in Golgi biogenesis.

The added discussion of the T301A result is also helpful. The finding that only a small fraction of KIN-G is phosphorylated at Thr301 in asynchronous cells makes the lack of a strong T301A phenotype more understandable.

Overall, I am happy with the revision of the beautiful manuscript.

Thank you for your critical review and insightful comments.

**Reviewer #2 (Public review):**

Summary:

The authors identify KIN-G as an in vitro substrate for phosphorylation by TbPLK and show that several of the in vitro P-ated sites, including T310, overlap with P-ation sites seen in live cells. The authors further show that PLK-mediated P-ation inhibits KIN-G binding to microtubules in vitro, as does a KIN-G-T301D mutant, and that expression of a KIN-G-T301D Phospho-mimic in *T. brucei* phenocopies KIN-G RNAi knockdowns, producing defects in cell division, morphogenesis of the centrin arm, FAZ and other cellular structures, as well as misplaced cytokinesis furrow.

Understanding cytoskeletal rearrangements that drive cell division in *T. brucei* is an important and unresolved problem, so the work addresses important questions that are of great interest. PLK and KIN-G have previously been shown to be important for cell division and morphogenesis of cytoskeletal structures that drive cell division in *T. brucei*. The current work advances our understanding by suggesting a potential mechanism by which PLK and KIN-G might participate, namely through PLK-dependent P-ation to control KIN-G MT binding activity.

Strengths:

The authors use a rigorous combination of biochemistry, phosphoproteomics, cell biology, and mutant analysis to support their conclusion that PLK-mediated P-ation of KIN-G negatively regulates KIN-G microtubule binding and this may explain the observation that a KIN-G T301 phosphomimic mutant blocks cell division and perturbs biogenesis of cytoskeletal structures that drive cell division and morphogenesis. Combining rigorous and informative in vitro studies with mutant analysis in live cells is a great strength. The work is solid and important, though a few pieces are needed to fully connect the in vitro findings with the in vivo observations, as detailed below.

Weaknesses:

Overall, I find this work to be solid, and to provide an important addition to our understanding of mechanisms controlling cell division in *T. brucei*. The biochemistry, in particular, is rigorous and convincingly demonstrates PLK can P-ate KIN-G, altering its MT-binding ability. Analysis of phospho-mutants of KIN-G in live *T. brucei* support the conclusion that P-ation of KIN-G at T301 negatively affects KIN-G function in vivo. I think, however, that the results fall short of supporting the title, because, although the data convincingly show that PLK can phosphorylate KIN-G at T301 in vitro, and that T301 is P-ated in vivo, they do formally demonstrate (nor even test) whether PLK is the kinase responsible for this phosphorylation in vivo (experiments to address this seem quite feasible). I also do not see where the authors try to reconcile the absence of phenotype for KIN-G-T301A with the implied importance of KIN-G phosphorylation by PLK in cell division, which calls into question the need for P-ation of KIN-G-T301 in cell division. Suggestions for addressing these concerns are provided below.

My two main questions are:

(1) What is the biological relevance of KIN-G P-ation at T301?

(a) The authors report no defect for the KIN-G-T301A mutant, so what then is the need for T301 P-ation, if the cell gets along fine without it? One step toward addressing this would be to ask what fraction of KIN-G shows P-ation at T301. Although published studies indicate P-ation at T301, it isn't known what percentage of KIN-G in the cell is P-ated. One might anticipate, for example, that T301-P is a small minority of the population in asynchronous cultures and that T301 P-ation increases at specific cell cycle stages.

(b) Published work links PLK to cell division, FAZ elongation, etc... The current work suggests that one role of PLK is to P-ate KIN-G at T301. In contrast, however, the current work also indicates that P-ation of KIN-G at T301 is unnecessary for normal cell division, FAZ elongation, etc....

(c) Some experiments or at least commentary on points a and b above would strengthen the paper.

- The authors have now addressed this question by assessing what % of KING is phosphorylated at T301 and adding commentary on this point in the revised paper.

- I would suggest that the model (new figure 8) include a dephosphorylation step, as that is proposed by the authors in the text. Also include in the legend some commentary on the role of phosphorylation, which is the center point of this paper, but not currently mentioned.

We have modified the model in Figure 8 to include dephosphorylation by an unknown protein phosphatase and a statement about the role of TbPLK phosphorylation on KIN-G function. Thank you.

(2) Is PLK the kinase that P-ates Kin-G T301 in vivo?

(a) The authors show PLK P-ates T301 (and other residues) in vitro, and that T-301 is P-ated in vivo. To bring the analysis full circle, it would be informative to examine KIN-G P-ation in a PLK mutant or upon inhibition of PLK with published inhibitors. This seems to be a very doable experiment with the tools available.

- The authors have addressed this question by demonstrating that T301 phosphorylation is reduced upon treatment with a PLK inhibitor, thus supporting that PLK phosphorylated T301 in vivo. It is noted that one might consider an alternate kinase is also able to phosphorylate T301 in absence of PLK activity, as that could explain the relatively low (~27%) reduction in phosphorylation by PLK inhibitor treatment.

Thank you.

**Reviewer #3 (Public review):**

Summary:

Here the authors investigate the role of the *Trypanosoma brucei* polo-like kinase TbPLK in the function of flagellum-associated cellular structures in trypanosomes. They set out to test the hypothesis that a key substrate of TbPLK is the kinesin protein KIN-G, and that TbPLK phosphorylation of KIN-G regulates its functions in cells.

Strengths:

Using in vitro biochemistry with purified proteins, the authors convincingly demonstrate that TbPLK phosphorylates KIN-G at 29 sites. Moreover, they convincingly show that phosphorylation at one site, T301, impairs the binding of purified KIN-G to purified microtubules. They further confirm that inhibition of TbPLK in cells reduces KIN-G phosphorylation at T301 (and S569). Using immunofluorescence-based imaging approaches, they also show that TbPLK colocalizes with KIN-G at centrin arms during early S-phase of the cell cycle. Centin arms are structures that are located near the basal body and flagellum and are important for new flagellum biogenesis, Golgi positioning, and cell division. To evaluate the function of KIN-G phosphorylation in cells, they depleted KIN-G by RNAi, simultaneously expressed phospho-mimetic (T301D) and phospho-ablative mutant proteins, and used immunofluorescene to examine the impact on flagellum-associated cellular structures. They show that expression of the phospho-mimetic mutant KIN-G-T301D causes the following defects: reduced cell proliferation, disruption of centrin arm and Golgi biogenesis, impairment of FAZ elongation and flagellum positioning, and misplacement of the cell division plane. The data convincingly support the conclusion that KIN-G phosphorylation on T301 plays an important role in regulating the cellular functions of this kinesin motor protein.

Weaknesses:

The authors have addressed prior weaknesses in the manuscript through additional experimentation and rewording of the conclusions.

Thank you for your critical review of our manuscript and for the very constructive comments and suggestions to improve the manuscript.

**Recommendations for the authors:**

**Reviewer #2 (Recommendations for the authors):**

(There is some redundancy below with my comments in the public review, but I've included here for clarity and further explanation.)

The authors have addressed my primary concern, as treatment with PLK inhibitor reduces phosphorylation of T301, while also providing some comment on relative impact of PLK-mediated KIN-G phosphorylation.

It is notable that phosphorylation of T301 was reduced by only ~27%, while phosphorylation of S569 was reduced by ~100% in the presence of PLK inhibitor. The authors note that this might be explained by slower dephosphorylation of T301. In the absence of a phenotype, and with cell doubling continuing unabated in presence of the inhibitor, it is intriguing that more loss is not observed. An alternative explanation is that an alternate kinase might also be able to phosphorylate T301 in the absence of PLK activity, and the authors should consider that possibility.

We added a sentence in the main text to suggest an alternative explanation.

The model shown in figure 8 should include a dephosphorylation step, per the authors comments in the text regarding the small fraction of T301 that is phosphorylated and proposal of a phosphorylation/dephosphorylation cycle. The Fig 8 legend needs to have some commentary on the role of phosphorylation, as phosphorylation is the center point of this paper.

We have modified the model in Figure 8 to include dephosphorylation by an unknown protein phosphatase and a statement about the role of TbPLK phosphorylation on KIN-G function.

Minor comments for improving the text are:

(1) The paper overall is clearly written. However, the Discussion starts with a solid sentence, then becomes a bit diffuse in discussing a wide range of PLK activities that were not addressed in the current work. That detracts attention a bit from the central contributions of this paper.

(2) At least two places in the text state apparent contradictions.

(a) p.5 and Fig 2C. The authors say microtubule gliding speed was "...insignificantly reduced..." by the TbPLK-K70R mutant, yet they then state that motility was "interfered with". If the effect is "insignificant", why do they claim there is an effect?

(b) p6 and Fig 3C. The authors report KIN-G-T301A impact on microtubule gliding activity is insignificant, but then say this mutation reduces motility of KIN-G. These statements are contradictory.

(3) p. 8, and Fig 7. "ventral side" and "leading edge" are not defined but are used to describe the KIN-G RNAi phenotype.

(4) Fig 7B. Please explain labeling - the new flagellum daughter is indicated as having the old posterior, while the old flagellum daughter cell is indicated as having the new cell posterior. This is counterintuitive to a reader not intimately familiar with the *T. brucei* cell division process.

(5) Fig 4, 5, and 7: "% Cells" is reported. Please indicate what number of cells total were examined.

These minor comments have already been addressed in the previous revision.
